# The transmissibility of novel Coronavirus in the early stages of the 2019-20 outbreak in Wuhan: Exploring initial point-source exposure sizes and durations using scenario analysis

**DOI:** 10.12688/wellcomeopenres.15718.1

**Published:** 2020-02-03

**Authors:** Sam Abbott, Joel Hellewell, James Munday, CMMID nCoV working group, Sebastian Funk

**Affiliations:** 1 Center for the Mathematical Modelling of Infectious Diseases, London School of Hygiene & Tropical Medicine, London, WC1E 7HT, UK

**Keywords:** coronavirus, outbreak, wuhan, modelling, transmission

## Abstract

**Background**: The current novel coronavirus outbreak appears to have originated from a point-source exposure event at Huanan seafood wholesale market in Wuhan, China. There is still uncertainty around the scale and duration of this exposure event. This has implications for the estimated transmissibility of the coronavirus and as such, these potential scenarios should be explored.

**Methods**: We used a stochastic branching process model, parameterised with available data where possible and otherwise informed by the 2002-2003 Severe Acute Respiratory Syndrome (SARS) outbreak, to simulate the Wuhan outbreak. We evaluated scenarios for the following parameters: the size, and duration of the initial transmission event, the serial interval, and the reproduction number (R0). We restricted model simulations based on the number of observed cases on the 25th of January, accepting samples that were within a 5% interval on either side of this estimate.

**Results**: Using a pre-intervention SARS-like serial interval suggested a larger initial transmission event and a higher R0 estimate. Using a SARs-like serial interval we found that the most likely scenario produced an R0 estimate between 2-2.7 (90% credible interval (CrI)). A pre-intervention SARS-like serial interval resulted in an R0 estimate between 2-3 (90% CrI). There were other plausible scenarios with smaller events sizes and longer duration that had comparable R0 estimates. There were very few simulations that were able to reproduce the observed data when R0 was less than 1.

**Conclusions**: Our results indicate that an R0 of less than 1 was highly unlikely unless the size of the initial exposure event was much greater than currently reported. We found that R0 estimates were comparable across scenarios with decreasing event size and increasing duration. Scenarios with a pre-intervention SARS-like serial interval resulted in a higher R0 and were equally plausible to scenarios with SARs-like serial intervals.

## Introduction

The ongoing outbreak of novel Coronavirus appears to have originated from an initial point-source exposure event at Huanan seafood wholesale market in Wuhan, China, which was closed on the 31st of December 2019 ^1, 2^. As of the 26th of January 2020 there have been over 2000 confirmed cases with the majority in China ^3^. Globally, countries are on high alert, with wide implementation of airport checks and contact tracing find and quarantine infected individuals. In China, officials have restricted travel across a wide area. There is still uncertainty around the precise scale and duration of the initial exposure event ^4^. The nature of the initial exposure has implications for estimates of the transmissibility of the coronavirus, as such it is important that these potential scenarios are further explored.

We used a stochastic branching process model to simulate the Wuhan outbreak, parameterised with available data where possible and otherwise informed by outbreaks of other coronaviruses, such as the 2002–2003 outbreak of Severe Acute Respiratory Syndrome Coronavirus (SARS-CoV) and multiple outbreaks of Middle East Respiratory Syndrome Coronavirus (MERS-CoV). We considered a realistic range of parameters where data were not available, quantifying how likely these scenarios were to occur using reported cases. We focused on the size and duration of the initial exposure event in particular, and the impact that this has on the estimated level of human-to-human transmission. We aimed to provide decision makers, and researchers, with probability estimates for each scenario considered, along with estimates of the reproduction number (R0) across all scenarios.

## Methods

### Branching process model

We modelled the outbreak using a stochastic branching process model comparable to those used elsewhere to model the dynamics of this outbreak ^4^. We assumed that cases from the initial transmission event were uniformly distributed over the duration of the event. Each case then resulted in a subsequent generation of cases with the number of cases that each case generated being drawn from a negative binomial distribution, to account for overdispersion, with a dispersion parameter k of 0.16 (assuming SARS-like dispersion) ^5^. The mean number of cases generated by each case (R0) was sampled from a uniform distribution once per model simulation with a lower and upper bound determined by the scenario being evaluated. New generations of cases were then sampled iteratively until the maximum simulation time was reached. We used three scenarios for the serial interval distribution informed by previous outbreaks of coronaviruses: SARS-like, with a mean of 8.4 days and standard deviation of 3.8 days ^5^; SARS-like before interventions, with a mean of 10 days and standard deviation of 2.8 days; and MERS-like, with a mean of 6.8 days and standard deviation of 4.1 days ^6^. Both SARS-like serial interval scenarios used a Weibull distribution, whilst the MERS-like serial interval scenario used a Gamma distribution ^5, 6^. After the simulation of the branching process, reporting delays were added as reported in a line-list of cases compiled from media and other reports ^7^. We fitted a geometric, Poisson, and a negative binomial distribution to these observed delays and selected the best fit using the Chi-squared statistic. If no good fit was determined using a p-value threshold of 0.05, then the reporting delay was instead sampled from the empirical delays in the line-list.

### Scenario analysis

We simulated the branching process model 10,000 times for all combinations of the following parameters: number of confirmed cases resulting from the initial exposure (20, 40, 60, 80, 200, 400), initial exposure event duration (1 day, 7 days, 14 days, 21 days, and 28 days), the serial interval distribution (SARS-like, initial SARS-like and MERS-like), and R0 (lower and upper bounds of a uniform distribution: 0-1, 1-2, 2-3, 3-4). We ran the model from the beginning of the outbreak for each scenario until the 25th of January 2020. The start date was determined by combining the duration of the transmission event with the date the fish market in Wuhan, the source of the outbreak, closed (31st of December 2019). We evaluated the samples from each scenario based on how closely their trajectories matched the 1,975 confirmed cases observed on the 25th of January ^7^. Samples were rejected if their simulated cumulative case estimates were outside a 5% interval on either side of this (1,876 - 2,074). Outbreak simulation was stopped if a sample exceeded the upper bound on the number of observed cases.

### Analysis

We visually compared the percentage of samples that were accepted for each combination of transmission event size, transmission event duration, mean serial interval, and R0 using a heat map. We then compared the distribution of R0 for accepted samples by transmission event size, transmission event duration and mean serial interval. We reported 90% credible intervals (CrI) for R0, stratified by the transmission event size, transmission event duration and the assumed mean serial interval.

### Implementation

All analysis was carried out using R version 3.6.2 ^8^. The branching process model was implemented using the bpmodels 0.1.0 package ^9^. The analysis is available as an open-source R package ^10^. A dockerfile has been made available with the code to ensure reproducibility ^11^.

## Results

### Percentage of outbreak simulations accepted

**Figure 1.**
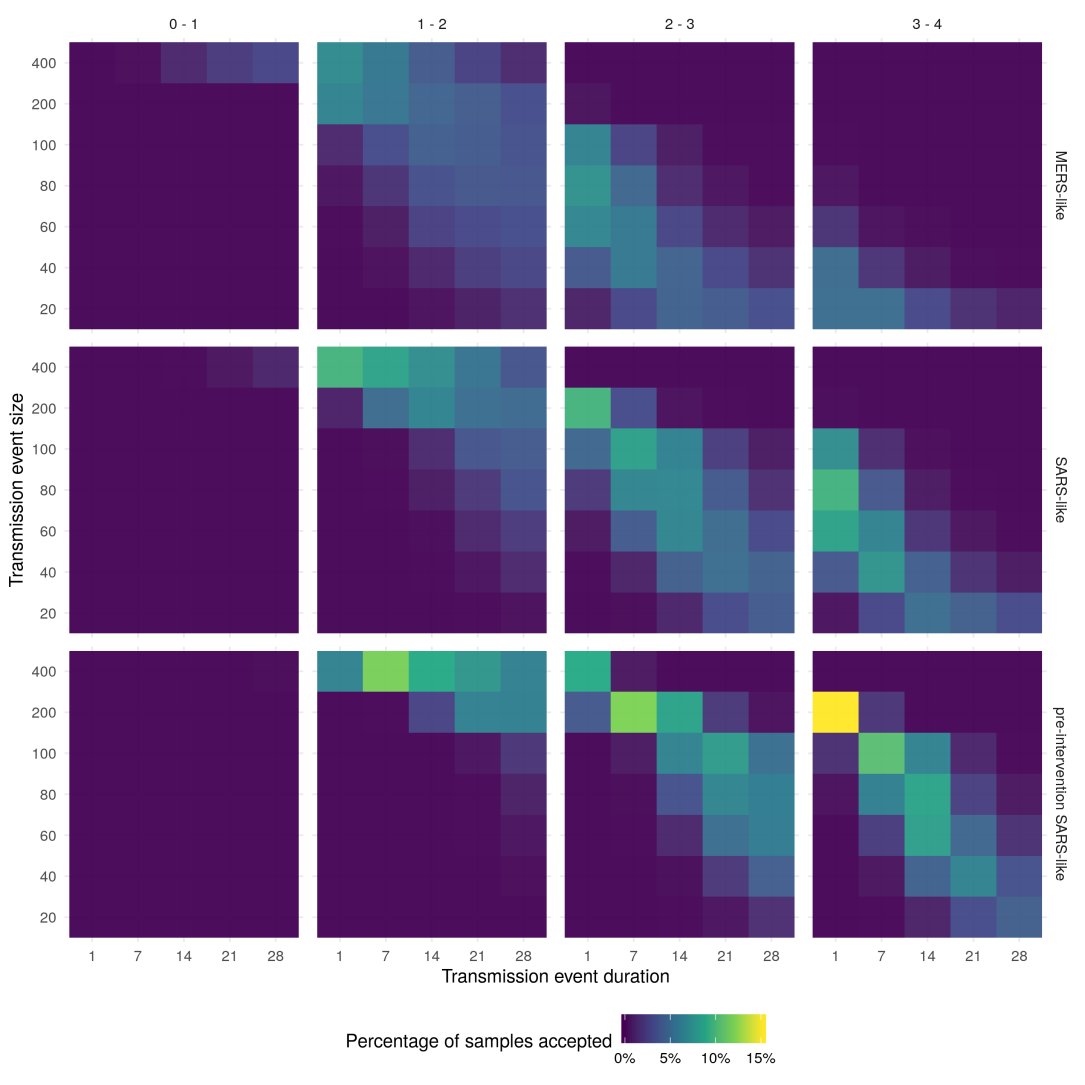
Heatmaps of the percentage of samples accepted for each combination of parameters. Within each heatmap, the x-axis represents the duration of the initial seeding event and the y-axis represents the size of the initial seeding event. The figure is stratified by the R0 scenario (columns) and the serial interval distribution (rows).

Overall, the highest acceptance rate was for scenarios with a large event size (200), short duration (1 day), an R0 between 3 and 4, and a pre-intervention SARS-like serial interval ( Figure 1). Scenarios with a SARS-like serial interval, an R0 bounded between 2 and 3, a short duration, and a relatively large event size (100) also had a high acceptance rate. Across all scenarios a higher acceptance rate was correlated with a larger event size, a shorter event duration, and a larger mean serial interval. This may be related to the influence these parameters have on the degree of volatility in outbreak simulations. Based on this, trends in Figure 1 should be interpreted with care using prior knowledge. For example, if the event size, serial interval, and event duration is assumed, then the percentage of acceptance may be used to infer the most likely R0 scenario.

There were very few scenarios where an R0 smaller than 1 resulted in scenarios that were accepted after conditioning on observed data, this was true regardless of the corresponding serial interval distribution, event size, or event duration. A very large event size (400) was required for scenarios with an R0 upper bound of 2 to have a moderate percentage of samples accepted if they had a short duration. Acceptance rates increased as the duration of the initial transmission event increased, and as the mean serial interval increased. For a MERS-like serial interval, the percentage of accepted samples was low for all scenarios, with the highest accepted proportion for scenarios with an upper bound on the R0 of 3 and a moderate event size, or an R0 upper bound of 2 and a larger event size.

### Estimated reproduction numbers

**Figure 2.**
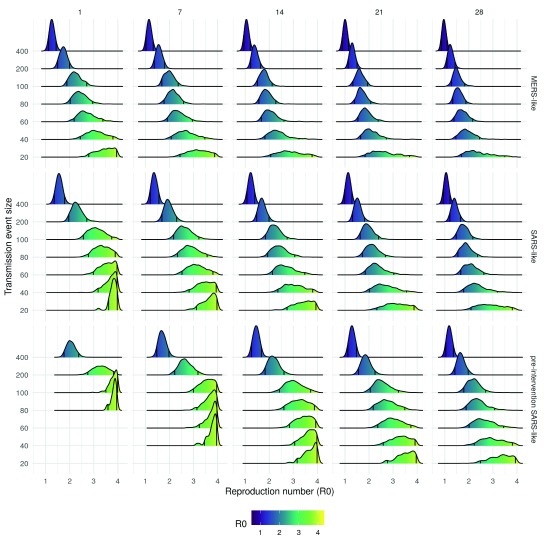
Density plot of reproduction number (R0) estimates from each accepted sample stratified by transmission event size, event duration (columns), and the serial interval distribution used (rows). The black lines on each density plot represent the 90% credible interval.

Uncertainty in the R0 estimate increased both as the event size decreased, and decreased as the mean serial interval increased ( Figure 2). Large event sizes resulted in the lowest R0 estimates across all scenarios evaluated. The estimated R0 decreased as the event size decreased and duration increased for all serial interval scenarios ( Table 1, Table 2, and Table 3). The most likely scenario with a MERS-like serial interval had an event size of 80 and a duration of a day, resulting in an estimated R0 between 2 – 3 (90% CrI, Table 1). For the SARS-like interval the most likely scenario had an event size of 200 and a duration of a day ( Figure 1), this resulted in an estimated R0 between 2 – 2.7 (90% CrI, Table 2). The most likely scenario with a pre-intervention SARS-like serial interval also had an outbreak size of 200 and a duration of a day, resulting in an estimated R0 between 2.8 - 3.8 (90% CrI, Table 3). Assuming a MERS-like serial interval resulted in an approximate decrease of 0 - 0.5 in the R0 estimates across all scenarios when compared to the SARS-like serial interval. Assuming a pre-intervention SARS-like serial interval resulted in an approximate increase of 0.5 - 1 in the R0 estimates across all scenarios when compared to the SARS-like serial interval. Across all serial interval scenarios R0 estimates were comparable when event size was decreased and event duration was increased in tandem.

**Table 1.** Estimated reproduction numbers (90% credible intervals) for the Wuhan outbreak conditioned on case data from the 25th of January, for scenarios with a MERS-like serial interval. Stratified by initial transmission event size and duration.

Transmission event size	Transmission event duration (days)				
	1	7	14	21	28
20	2.8 - 4	2.4 - 3.9	2.1 - 3.8	1.8 - 3.7	1.7 - 3.5
40	2.4 - 3.8	2.1 - 3.5	1.8 - 3.2	1.7 - 2.7	1.5 - 2.4
60	2.2 - 3.4	1.9 - 3	1.7 - 2.6	1.5 - 2.4	1.4 - 2.2
80	2 - 3	1.8 - 2.6	1.6 - 2.3	1.4 - 2.1	1.3 - 1.9
100	1.9 - 2.7	1.7 - 2.4	1.5 - 2.1	1.3 - 1.9	1.3 - 1.8
200	1.5 - 2	1.4 - 1.8	1.2 - 1.6	1.1 - 1.5	1.1 - 1.4
400	1.1 - 1.4	1 - 1.3	0.9 - 1.2	0.9 - 1.1	0.9 - 1.1

**Table 2.** Estimated reproduction numbers (90% credible intervals) for the Wuhan outbreak conditioned on case data from the 25th of January, for scenarios with a SARS-like serial interval. Stratified by initial transmission event size and duration.

Transmission event size	Transmission event duration (days)				
	1	7	14	21	28
20	3.6 - 4	3.1 - 4	2.7 - 3.9	2.3 - 3.9	2.1 - 3.8
40	3.2 - 4	2.8 - 3.9	2.4 - 3.8	2 - 3.6	1.8 - 3.2
60	3 - 4	2.5 - 3.8	2.1 - 3.5	1.8 - 3	1.7 - 2.6
80	2.8 - 3.9	2.3 - 3.6	1.9 - 3.1	1.7 - 2.7	1.5 - 2.4
100	2.6 - 3.7	2.2 - 3.2	1.8 - 2.7	1.6 - 2.4	1.5 - 2.2
200	2 - 2.7	1.7 - 2.3	1.5 - 2	1.3 - 1.8	1.2 - 1.7
400	1.4 - 1.8	1.2 - 1.6	1.1 - 1.4	1 - 1.3	0.9 - 1.2

**Table 3.** Estimated reproduction numbers (90% credible intervals) for the Wuhan outbreak conditioned on case data from the 25th of January, for scenarios with a pre-intervention SARS-like serial interval. Stratified by initial exposure event size and duration.

Transmission event size	Transmission event duration (days)				
	1	7	14	21	28
20	-	3.8 - 4	3.2 - 4	2.8 - 4	2.5 - 3.9
40	-	3.5 - 4	3.1 - 4	2.6 - 3.9	2.2 - 3.8
60	4 - 4	3.2 - 4	2.8 - 3.9	2.4 - 3.7	2 - 3.4
80	3.6 - 4	3.1 - 4	2.6 - 3.9	2.2 - 3.5	1.9 - 3.1
100	3.5 - 4	3 - 4	2.4 - 3.7	2.1 - 3.2	1.8 - 2.7
200	2.8 - 3.8	2.2 - 3.2	1.8 - 2.6	1.6 - 2.2	1.4 - 2
400	1.8 - 2.4	1.5 - 2	1.3 - 1.7	1.1 - 1.5	1 - 1.4

## Discussion

In this study, we explored a range of scenarios for the initial event size and duration of the exposure event which initiated the 2019–20 Wuhan novel coronavirus outbreak. We conditioned on observed cases to establish the probability of each scenario, given our model, and then estimated the R0 of coronavirus from the accepted simulations. We found that there was a very low probability that the reproduction numbers was less than 1 for any scenario considered. Across all serial interval scenarios larger exposure events over a shorter time horizon were most plausible. The most probable SARS-like serial interval scenarios resulted in an estimated R0 of 2 - 2.7 (90% CrI), whilst the most probable pre-intervention SARS-like serial interval scenarios resulted in an estimated R0 of 2.8 - 3.8 (90% CrI). MERS-like serial interval scenarios were less plausible, but the most plausible resulted in an estimate R0 of 2 - 3 (90% CrI). Reducing the event size led to estimates of the R0 increasing but also reduced the proportion of samples accepted. Similarly, increasing the event duration reduced the estimated R0 whilst decreasing the proportion of accepted samples. Decreasing the event size whilst increasing the duration resulted in R0 estimates that were comparable to those from the most plausible scenarios and reduced the acceptance rate the least.

Our study used a stochastic model to capture the transmission dynamics of the outbreak with parameters informed from data were possible, if there was no data available then parameters were assumed to be similar to those estimated for SARS ^5^. We only fitted to the cumulative data at one time point, on 25 January 2020, as time-resolved data of onsets was not available at this point in time. It has also been reported that it is likely that the efforts to confirm suspected cases have changed over time, which also precludes fitting to earlier data points.

As the outbreak progresses time-resolved data of reported cases or disease onsets are likely to become available, with sufficiently consistent data reporting it is likely that other approaches will become superior to the one presented here. More data on the serial interval distribution, on variability of transmission and possible superspreading events, as well as on the timing and impact of interventions, is likely to become available during the course of the outbreak. This will make it possible to estimate the R0 with greater precision with less risk of bias due to unknown parameters. The number of scenarios that need to be evaluated may also be reduced as additional information about cases connected to the initial exposure event becomes available. Though our estimates had wide credible intervals it is possible that we could not fully account for the numerous sources of bias and uncertainty present in the available data. This means that our model estimates may be both spuriously precise and potentially biased. There is some evidence of this in our results as the scenarios with the highest acceptance rate were on the edge of our scenario grid both for event size, event duration, and mean serial interval. This may be the result of these scenarios reducing volatility and therefore having narrower distributions of estimated cases. Indeed, we found that R0 estimates were comparable as event size decreased and event duration increased. Expert knowledge relating to the size and duration of the initial event may help clarify this issue. Alternatively, other estimates of R0 may be used to indicate which event size and event duration scenarios are most plausible.

A previous study also looked at varying the event size and the impact that this had on R0 estimates using a branching process ^4^. Our work builds on this by also looking at event duration, including reporting delays, and using a different approach to condition on observed cases. For comparable scenarios, our results were similar to those previously published but we found that R0 estimates were highly sensitive to variation in the assumed serial interval, event size, and event duration. We made use of a highly reproducible framework (an R package) and have released all of our code as open-source ^10^. This means that this analysis may be repeated - both by the authors and others - as more data becomes available. In addition, subject area experts may be able to adapt our analysis using this open-source code to reduce the potential for bias using their expert knowledge or privately held data.

The R package we have developed alongside our analysis may be generalisable to other point source outbreaks when time series data on cases is unavailable or difficult to verify. Additional work is needed to ensure the robustness of this tool but this may allow this analysis to be repeated during future outbreaks with little additional overhead.

This analysis used a stochastic branching process to explore scenarios around the duration and size of the initial exposure event at the Huanan seafood wholesale market in Wuhan. Despite the scarcity of data currently available our estimates may be used to rule out some scenarios and to assess the likelihood of others. Our results indicate that it is very unlikely that the infectious agent responsible for the Wuhan outbreak has a R0 of less than 1, unless the size of the transmission event was much greater than currently reported. We also found that a large initial exposure event was likely, combined with a short duration. These scenarios resulted in R0 estimates that are comparable to those estimated during the 2002–2003 SARS outbreak. However, with the available data we could not identify whether scenarios with a SARS-like or pre-intervention SARS-like serial interval were more likely. As more information becomes available it may be possible to further refine our results and establish the value of R0. Providing clear quantitative information for decision makers on the transmissibility of coronavirus is of clear public health importance. Our work to make this process reproducible may reduce the time these estimates take to be made available in future outbreaks and increase knowledge sharing across response teams.

## Data availability

### Underlying data

Zenodo: epiforecasts/WuhanSeedingVsTransmission: Resubmission to Wellcome Open. https://doi.org/10.5281/zenodo.3631830 ^10^

This project contains the following underlying data:

- inst/results/grid.fst (The complete results of our scenario analysis)
- inst/results/conditioned_grid.fst (The results of our scenario analysis conditioned on observed cases)
- inst/results/proportion_sims_allowed.fst (The proportion of samples allowed per scenario evaluated)
- data/fitted_delay_sample_func.rda: (This is a reporting delay function as discussed in the text)

Data is available alongside the source code under the terms of the MIT License.

## Software availability

Source code is available from: https://github.com/epiforecasts/WuhanSeedingVsTransmission/tree/v0.3.0

Archived source code at time of publication: http://doi.org/10.5281/zenodo.3631830 ^10^

License: MIT
