# Supplementary material for: The transmissibility of novel Coronavirus in the early stages of the 2019-20 outbreak in Wuhan: Exploring initial point-source exposure sizes and durations using scenario analysis
Source: Wellcome Open Res. 2020 Feb 3;5:17. [Version 1] doi: 10.12688/wellcomeopenres.15718.1 (PMC7156988; doi:10.12688/wellcomeopenres.15718.1)
Supplement: Peer review history

# Peer review: Reviewer response for version 1

**DOI:** 10.21956/wellcomeopenres.17230.r38320

**Published:** 2020-04-14

**Authors:** Andrei R. Akhmetzhanov

**Affiliations:** 1 Graduate School of Medicine, Hokkaido University, Sapporo, Japan

In my opinion, the analysis of possible scenarios for early dynamics of the outbreak can be both quite interesting and helpful for the future. In this sense I enjoyed reading the manuscript. However, I have had several remarks to the authors and it would be great to clarify them.

*Scenario analysis*

- One of the exposure event duration was taken 28 days. Could the authors give some motivation for this long exposure period? (for example, by one-two sentences in their introduction).
- My main concern comes from the fact that the ending time (31st of December 2019) was fixed in the simulation, but the starting moment was in range between 1–28 days. In my opinion this scenario misses the very important one: what if there was a short-term exposure around the end of November – beginning of December that followed by a human-to-human transmission chain? At least, the epicurve published earlier in Lancet might suggest such scenario, and also an unofficial article published in FT proposed something similar. In this regard I would also recommend to consider the fixed starting time, but varied length of the exposure. I wonder if the conclusions of the authors could be changed in this case.
- The transmission event size looks a bit large, and I had the feeling that the authors was a bit biased in their desire to obtain R0 around 2-3. In this, regard, why not to provide some information on R0 estimates for small enough values of the event size. For example, if I consider, say, the size of 5 or size of 2? How unlikely would be the resulting estimate? I think it could be possible to have also big values of R0 at the beginning.

Is the work clearly and accurately presented and does it cite the current literature?

Yes

If applicable, is the statistical analysis and its interpretation appropriate?

Yes

Are all the source data underlying the results available to ensure full reproducibility?

Yes

Is the study design appropriate and is the work technically sound?

Yes

Are the conclusions drawn adequately supported by the results?

Yes

Are sufficient details of methods and analysis provided to allow replication by others?

Yes

Reviewer Expertise:

Mathematical modeling of infectious disease outbreaks

I confirm that I have read this submission and believe that I have an appropriate level of expertise to confirm that it is of an acceptable scientific standard.

# Peer review: Reviewer response for version 1

**DOI:** 10.21956/wellcomeopenres.17230.r37777

**Published:** 2020-02-11

**Authors:** Christian Althaus

**Affiliations:** 1 Institute of Social and Preventive Medicine (ISPM), University of Bern, Bern, Switzerland

The study by Abbott and colleagues is one of several 2019-nCoV modelling studies that have appeared during the last two weeks and describes the early transmission dynamics during the outbreak in Wuhan, China. Using a stochastic branching process model, the authors find that the observed outbreak is most compatible with a large initial transmission event, a SARS-like serial interval, and an R0 between 2 and 4. What I particularly like about the study - and what distinguishes it from some of the other modelling papers - is the addition of reporting delays to the simulated case counts.

 While the modelling approach is sound, a couple of things about the simulation procedure remain somewhat unclear:

1. The authors write that "The start date was determined by combining the duration of the transmission event with the date the fish market in Wuhan, the source of the outbreak, closed (31st of December 2019)." How exactly was the start date determined? The start date is actually one of the most crucial aspects of the analysis, and it is not reported in the text.
2. The authors write that they either use a parametric description of the reporting delay or sample from the empirical delays. Does this mean that they use different delays  at different times during the outbreak?

Overall, I think that the study would gain from discussing the recent literature on the outbreak dynamics and findings from phylogenetic analyses. In particular, I think the authors should discuss their findings in light of the studies by Riou & Althaus ^1^ (Euro Surveill, PMID: 32019669) and Li *et al.* ^2^ (N Engl J Med, PMID: 31995857) which provided the first published estimates of R0 for 2019-nCoV. It would also be interesting to compare the start date of the simulated outbreaks to preliminary estimates of tMRCA (see http://virological.org, for example). Finally, I think the issue of under reporting and potential biases in using reported case counts deserves some additional discussion.

 Minor point:

1. In the Abstract, it is maybe somewhat unclear to a non-expert what the authors mean by SARS-like and pre-intervention SARS-like serial interval.

Is the work clearly and accurately presented and does it cite the current literature?

Partly

If applicable, is the statistical analysis and its interpretation appropriate?

Not applicable

Are all the source data underlying the results available to ensure full reproducibility?

Yes

Is the study design appropriate and is the work technically sound?

Yes

Are the conclusions drawn adequately supported by the results?

Yes

Are sufficient details of methods and analysis provided to allow replication by others?

Partly

Reviewer Expertise:

Infectious disease epidemiology

I confirm that I have read this submission and believe that I have an appropriate level of expertise to confirm that it is of an acceptable scientific standard.

## References

[1] : Pattern of early human-to-human transmission of Wuhan 2019 novel coronavirus (2019-nCoV), December 2019 to January 2020. *Euro Surveill*.2020;25(4) : 10.2807/1560-7917.ES.2020.25.4.2000058 10.2807/1560-7917.ES.2020.25.4.2000058 32019669PMC7001239

[2] : Early Transmission Dynamics in Wuhan, China, of Novel Coronavirus-Infected Pneumonia. *N Engl J Med*.2020; 10.1056/NEJMoa2001316 10.1056/NEJMoa2001316 31995857PMC7121484
